# Antimicrobial Protection of Marsupial Pouch Young

**DOI:** 10.3389/fmicb.2017.00354

**Published:** 2017-03-07

**Authors:** Yuanyuan Cheng, Katherine Belov

**Affiliations:** School of Life and Environmental Sciences, The University of Sydney, SydneyNSW, Australia

**Keywords:** marsupials, pouch microbiota, pouch young, marsupial milk, antimicrobial peptide, cathelicidin

## Abstract

Marsupials diverged from eutherian mammals about 148 million years ago and represent a unique lineage of mammals with distinctive morphological and reproductive characteristics. Marsupials have significantly shorter gestation periods than eutherians. Pregnancy typically ranges from 15 to 35 days, with young being born at a very early developmental stage and lacking differentiated lymphoid tissues and mature effector cells. Recent microbiome studies of the marsupial pouch revealed that marsupial young can face intense microbial challenges after birth, as the pouch contains a broad range of Gram-positive and Gram-negative bacteria. Antimicrobials are believed to play a significant role in the immune protection of marsupial newborns during their pouch life. The skin of the post-reproductive pouch secretes antimicrobial lysozyme and dermcidin, which may contribute to the decreased density of certain bacteria in the pouch. A range of antimicrobial agents, such as immunoglobulins, lysozyme, transferrin, and cathelicidins, have been identified in marsupial milk. Antimicrobial assays have revealed that marsupial cathelicidins have broad-spectrum activity against a variety of bacteria and fungi, including several multi-drug resistant strains. In this article, we will review the action mechanisms of these antimicrobial compounds and discuss how they protect marsupial newborns from potentially pathogenic bacteria inside the pouch. We will also discuss the potential of marsupial antimicrobial compounds as a source of novel antibiotics.

## Introduction

Marsupials last shared a common ancestor with eutherians around 148 million years ago (Bininda-Emonds et al., 2007) and represent a unique lineage of mammals with distinctive morphological and reproductive characteristics. There are over 300 extant marsupial species distributed in the Americas and Australasian regions, constituting about 7% of the world’s living mammals (Dickman, 2005). As one of the most diverse mammalian taxa, marsupials exhibit enormous variations in the body size, reproductive strategy, and other life history traits. The adult body mass ranges from less than 5 g in planigales (genus *Planigale*) to over 80 kg in the red kangaroo (*Macropus rufus*), and life span varies greatly from less than a year for males of several small dasyurid species to up to 27 years for large kangaroos (Dickman, 2005). Many herbivorous species have long inter-birth intervals (longer than 12 months) and produce one offspring per litter, whereas some insectivorous species have significantly higher reproductive rates with the litter size larger than 10 and the inter-birth interval shorter than 3 months (Fisher et al., 2001).

Despite the extensive diversity in distribution, diet, life history and ecology, marsupials share some common features which separate them from other mammals. All marsupials have short gestation periods (9–42 days) and give birth to highly underdeveloped young that weigh less than 1% of the mass of the mother (Dickman, 2005). Due to such short gestations, marsupial neonates lack developed immune tissues and mature lymphocytes, which makes them incapable of mounting adaptive immune responses (Basden et al., 1997; Old et al., 2004). Physical and immunological development takes place inside the mother’s pouch, a fold of skin on the abdomen that covers the teats. Some marsupials (caenoletids, some didelphids, and most dasyurids) do not have a fully developed pouch, but instead have rings of muscle in the skin surrounding the teats that temporarily contracts during lactation to provide cover for the young (Dickman, 2005). The pouch environment contains a large variety of microbes which can pose pathogenic treats to the altrical young (Deakin and Cooper, 2004; Chhour et al., 2010; Cheng et al., 2015). Recent research on how marsupial neonates survive the non-sterile environment of the pouch has shed light on the importance of non-specific components of the mammalian immune system in fighting infections. In this article, we will provide an overview of the pathogenic challenge faced by marsupial young during their development in the pouch, and discuss major mechanisms and key antimicrobial agents involved in pouch young protection.

## Microbiota in the Pouch

The pouch microbiota of marsupials has primarily been investigated in three model species – the tammar wallaby (*Macropus eugenii*), brushtail possum (*Trichosurus vulpecula*), and Tasmanian devil (*Sarcophilus harrisii*). Two earlier papers are also available for the koala (*Phascolarctos cinereus*) (Osawa et al., 1992) and quokka (*Setonix brachyurus*) (Charlick et al., 1981), both using culture-based techniques to examine bacterial strains inside the pouch. Here, we focus on data from more recent studies that utilized more sensitive molecular-based methods.

The tammar wallaby and brushtail possum both have a fully developed pouch that opens anteriorly (Tyndale-Biscoe, 2005). Deakin and Cooper (2004) explored the pouch of brushtail possums throughout the reproductive cycle via bacterial culture complemented by 16S rRNA gene sequencing. Among the 46 Gram-positive and 20 Gram-negative species isolated from 71 swabs, Gram-positive cocci species were suggested to be the most abundant with the opportunistic pathogen *Staphylococcus aureus* representing one of the most common members of the pouch bacterial community at all reproductive stages. Chhour et al. (2010) characterized the pouch flora of tammar wallabies by cloning bacterial 16S rRNA genes and sequencing isolates with unique restriction enzyme digestion patterns. A total of 41 phylotypes were identified in 227 clones from three pouch samples, among which Actinobacteria were detected as the predominant bacterial phylum accounting for 82.9% of total diversity. Several bacterial species that have been implicated in human or animal diseases were observed and the most notable was *Corynebacterium* spp. (such as *C. aurimucosum*, *C. macginleyi*, and *C. jeikeium*), which represented the most commonly isolated bacterial genus in the tammar wallaby pouch.

The Tasmanian devil’s pouch opens toward the rear. Sequencing of 16S rRNA gene amplicons on a Roche 454 GS FLX system revealed a highly diverse flora in the devil’s pouch with an average of 1,907 phylotypes (bacterial groups sharing > 97% sequence similarity in the 16S rRNA gene V1–V3 region) identified in each sample (Cheng et al., 2015). The observed microbiota was co-dominated by Firmicutes (36.2%) and Proteobacteria (34.4%), followed by Fusobacteria (9.8%), Bacteroidetes (7.0%), and Actinobacteria (3.3%). Of the detected Firmicutes, 77.1% were categorized to class Clostridia and 21.6% to Bacilli, while 88.6% of the Proteobacteria belonged to the Gamma subdivision. Several bacterial genera that contain significant human and animal pathogens were found to have high relative abundance in the devil’s pouch, such as Clostridium (9.5%), Fusobacterium (4.3%), Pseudomonas (4.2%), and Porphyromonas (2.8%).

All above evidence suggests that the marsupial pouch harbors a wide range of microbes which inevitably include pathogenic species. Additionally, studies also found that the female urogenital tract opening of the tammar wallaby (Chhour et al., 2008) and abdominal skin of the Tasmanian devil (Cheng et al., 2015) were colonized by various bacteria from five main phyla – Firmicutes, Bacteroidetes, Actinobacteria, Proteobacteria, and Fusobacteria. Bacteria at these sites can potentially be another source of infection for the immunologically naïve neonates while they crawl toward the pouch after birth. To cope with the pathogenic challenge, marsupials have evolved a range of defense strategies so the neonates can survive in the potentially hostile environment.

## Alteration of Pouch Environment During Lactation

**FIGURE 1:**
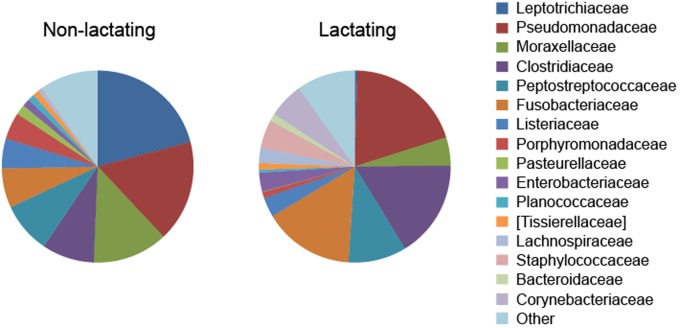
**Alteration of pouch microbiome during lactation in the Tasmanian devil (data from Peel et al., 2016)**.

Considerable alterations occur in the pouch flora when marsupials enter reproduction and lactation periods (Charlick et al., 1981), with lower levels of bacterial species richness found in the pouch when pouch young are present (Old and Deane, 1998; Deakin and Cooper, 2004; Chhour et al., 2010). A decrease in Gram-negative bacteria, such as *Klebsiella pneumoniae*, *Pseudomonas aeruginosa*, *Enterobacter aerogenes*, and *Escherichia coli*, was detected in the tammar wallaby pouch leading up to and right after (<6 days) parturition (Old and Deane, 1998). Female brushtail possums in anoestrus had a high proportion of Gram-positive cocci in the pouch, whereas those with pouch young had a lower proportion of such species (Deakin and Cooper, 2004). In the Tasmanian devil, a high degree of compositional dissimilarity was observed between the pouch microbiome in non-lactating and lactating females (**Figure 1**) (Peel et al., 2016). Several bacterial taxa that contain potentially pathogenic species showed significantly lower relative abundance in the pouch of lactating devils, for example, Leptotrichiaceae (reduced from 20.9% in non-lactating pouch to 0.4% in lactating pouch), Porphyromonas (4.5% down to 0.3%), Pasteurellaceae (1.7% down to 0.1%), and Parvimonas (1.0% down to 0.2%).

In regard to the mechanism underlying reproduction-associated alteration of pouch flora, it is believed that pouch skin secretions play a major role in changing the bacterial community profile. Pouch washes collected from koalas during breeding season were found to be inhibitory against *E. coli* and *Staphylococcus aureus* (Bobek and Deane, 2001). Similarly, pouch secretions of the tammar wallaby showed antimicrobial activity against *E. coli*, with the greatest inhibition of growth achieved using samples collected at the time of birth (Ambatipudi et al., 2008). A proteomic analysis of secreted proteins of the tammar wallaby and wombat (*Vombatus ursinus*) revealed that higher diversity of proteins was produced in the mature reproductive pouch than in the immature or post-reproductive pouch (Ambatipudi et al., 2007).

Among the 40 identified proteins secreted in the tammar wallaby and wombat pouch, lysozyme and dermcidin are two important compounds with known antimicrobial functions (Ambatipudi et al., 2007). Lysozyme is a powerful antibacterial protein that is present in a wide variety of animal secretions and fluids, such as milk, saliva, tears, and egg white. It was the first antimicrobial agent identified in human skin and is secreted by keratinocytes, sebocytes, hair bulb cells, and eccrine sweat glands (Schröder and Harder, 2006). Lysozyme is known to degrade the cell wall of Gram-positive bacteria via its muramidase activity, but can also disrupt the membranes of Gram-positive and Gram-negative bacteria in a non-enzymatic manner (Rene et al., 2003). Compared to lysozyme which has wide distribution, dermcidin is highly specialized in terms of tissue origin. It is exclusively and constitutively produced in the sweat glands and represents the dominant antimicrobial component in sweat (Schittek et al., 2001). Dermcidin and derived peptides exhibit broad-spectrum activity against bacteria and fungi, and have also been suggested to play a role in a range of cancers, such as adenocarcinoma and breast carcinomas (reviewed in Schittek, 2012). Interestingly, unlike most other host defense peptides (e.g., defensins and cathelicidins), dermcidin and derivatives do not rely on a positive net charge to exert their activity and their mode of action does not involve inducing membrane permeabilization in target cells (Steffen et al., 2006). These properties of lysozyme and dermcidin have enabled them to maintain activity over a broad range of pH and salt concentrations (Davies et al., 1969; Schittek et al., 2001). In light of this, although it is unclear what pH conditions are found in the marsupial pouch secretion, lysozyme and dermcidin likely play a significant part in regulating the pouch flora.

Another antimicrobial family that may contribute to pouch flora regulation are cathelicidins. A recent study found that cathelicidin genes are expressed in the skin and pouch lining in the Tasmanian devil (Peel et al., 2016). One cathelicidin (Saha-CATH2) showed the highest expression level in the pouch compared to 11 other tissues (including immune tissues such as lymph node and spleen). The role of cathelicidins in pouch young protection is further discussed in the following section.

Regulation through these antimicrobial agents not only leads to the decrease of potentially harmful bacteria in the pouch, it also results in elevated relative abundance of certain bacteria that may be beneficial for pouch young development. For example, the prevalence of Enterobacteriaceae was found to increase from 0.6% in non-lactating pouch to 2.9% in lactating pouch in the Tasmanian devil (Peel et al., 2016). This bacterial family contains several important lactic acid-producing species, such as *Lactobacillus* sp., which have been suggested to play key roles in maintaining healthy microbiota in the human vagina (reviewed in Eloe-Fadrosh and Rasko, 2013). Interestingly, the six tested Tasmanian devil cathelicidin peptides all showed low to no activity against *Enterococcus faecalis* strains (except for vancomycin-resistance *E. faecalis*), which belong to Enterobacteriaceae (Peel et al., 2016). These observations may indicate that certain members of this family act as symbiotic bacteria in the pouch environment. Symbiotic components of the pouch microbiota is one important subject that has not been adequately explored so far; such bacteria may play a crucial role in the protection and development of pouch young, and therefore require further investigation.

Although pouch secretions significantly reduce the prevalence of certain harmful microbes in the pouch during lactation, evidence found in the Tasmanian devil demonstrates that there are still a large variety of potentially pathogenic bacteria present in the lactating pouch (Peel et al., 2016). For example, several bacterial families that contain known human or veterinary pathogens showed high relative abundance in the pouch of lactating devils, including Pseudomonadaceae (19.7%), Clostridiaceae (16.4%), Fusobacteriaceae (15.3%), Corynebacteriaceae (6.0%), and Staphylococcaceae (4.8%) (**Figure 1**). Therefore, marsupials may still face intense pathogenic pressure after birth and further protective mechanisms are required for pouch young survival.

## Protection Through the Milk

**FIGURE 2:**
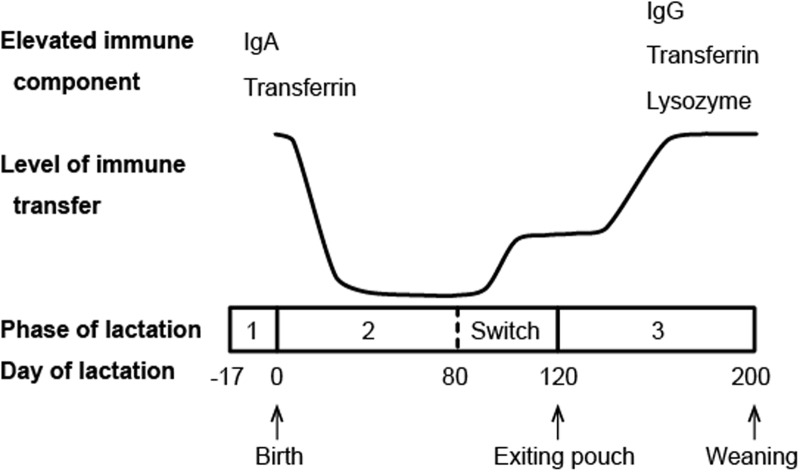
**Schematic diagram of immune transfer through milk in the brushtail possum (compiled from data presented in Demmer et al., 1998; Adamski and Demmer, 2000)**.

Another major channel of maternal immune protection for marsupial neonates is the milk. Compared to eutherian mammals, marsupials have a longer period of lactation, which can be divided into three phases (Tyndale-Biscoe, 2005). While phase 1, the preparation of the mammary gland before parturition, and phase 3, the growth phase of physiologically independent young, are equivalent to the characteristic lactation of eutherian mammals, phase 2 is unique to marsupials (**Figure 2**). Starting from the point when the neonate is attached to a teat and starts suckling, phase 2 lasts for a few months until the pouch young begins to generate its own body heat (Shaw, 2006). During this phase, the neonate is constantly protected in the pouch environment and obtains its nourishment solely from the milk. This is also the phase where the marsupial milk undergoes profound changes to meet the needs of rapidly developing pouch young (Tyndale-Biscoe, 2005).

Milk constituents involved in antimicrobial protection, such as immunoglobulins (Ig), lysozyme, transferrin, and host defense peptides, have been identified in multiple marsupial species. Most of these components are differentially expressed throughout lactation, in response to different developmental stages of the pouch young (Adamski and Demmer, 2000; Lefèvre et al., 2007; Wanyonyi et al., 2011).

Immunoglobulins (antibodies) are commonly found in mammalian milk with the relative abundance of different isotypes varying among species. Ingested antibodies provide the young not only immediate immunity, but also long term resistance against certain infections (Telemo and Hanson, 1996). In marsupials, a microarray study in the tammar wallaby and transcriptome analysis in the Tasmanian devil both showed that all four marsupial Ig isotypes (IgA, IgG, IgE, and IgM) are expressed in the milk (Daly et al., 2007; Hewavisenti et al., 2016). In the tammar wallaby, milk Ig expression exhibits two peak periods, with the first period occurring around parturition and the second around the end of phase 2 of lactation (Daly et al., 2007). Western blotting analysis of the brushtail possum milk also revealed two main periods of maternal Ig transfer (**Figure 2**), though the second peak does not appear until phase 3 of lactation (Adamski and Demmer, 2000). It is believed that these two periods of increased Ig transfer coincide with two developmental stages during which the pouch young is most immunologically vulnerable: immediately after birth when it has no adaptive immunity, and around the time when it emerges from the pouch and faces new pathogens in the environment (Adamski and Demmer, 2000).

Lysozyme is another key component of mammalian milk contributing to antimicrobial activity. It was found to be the most highly expressed immune gene in mid-lactation milk in the Tasmanian devil, accounting for 3.6% of all transcripts in the milk (Hewavisenti et al., 2016). In the brushtail possum, lysozyme has been isolated as a main component of the whey fraction of the milk (Piotte et al., 1997) and is continuously expressed in the mammary tissue throughout lactation (Demmer et al., 1998). By contrast, lysozyme in ringtail possum (*Pseudocheirus peregrinus*) milk appears to be a late lactation protein and is not detected until phase 3 (Nicholas et al., 1989). Interestingly, it has also been found that the concentration of lysozyme protein and derivatives in brushtail possum milk increases throughout lactation and reaches the highest expression level in phase 3 (Demmer et al., 1998; Kuy et al., 2007). These observations are indicative of the important role of marsupial lysozyme in protecting both mammary gland and pouch young against infection, especially when the young has left the pouch.

Transferrin, a family of iron-binding proteins responsible for iron storage and transport, has been identified in the milk of brushtail possums, tammar wallabies, and koalas (Adamski and Demmer, 2000; Lefèvre et al., 2007; Morris et al., 2016). These multi-functional proteins are also considered a component of innate immunity. Lactoferrin, a representative member of the transferrin family that is highly abundant in human milk, is known to have a wide range of antibacterial, antiviral, antifungal, and immunomodulatory activities (van Hooijdonk et al., 2007; Siqueiros-Cendon et al., 2014; Wakabayashi et al., 2014). The antimicrobial functions of marsupial transferrins have not been examined. However, the differential expression pattern of transferrin in brushtail possum milk mirrors that of antibodies, with the highest level of expression detected during the two major periods of immune transfer (**Figure 2**) (Adamski and Demmer, 2000), indicating that transferrin likely plays a role in pouch young protection.

Cathelicidins and defensins are two major families of host defense peptides in marsupials. These small cationic peptides function mainly by binding to and disrupting microbial cell membranes through electrostatic interactions, and many have potent broad-spectrum activities against a wide range of bacterial, fungal or viral pathogens (Ganz, 2003; Kościuczuk et al., 2012). So far, host defense peptides have been identified as a milk component in three marsupials via transcriptome analysis: four cathelicidins and three defensins were detected in milk of the Tasmanian devil, one cathelicidin was found in tammar wallaby milk, and four cathelicidins in koala milk (Lefèvre et al., 2007; Hewavisenti et al., 2016; Morris et al., 2016). Gene expression analysis of tammar wallaby cathelicidins in mammary gland demonstrated that similar to other immune components, cathelicidins are also differentially expressed throughout lactation (Wang et al., 2011; Wanyonyi et al., 2011). The mature peptides appeared to be most highly expressed during the early stage of phase 2, corresponding to the period when the neonate lacks mature adaptive immunity. Interestingly, Wanyonyi et al. (2011) also found that cathelicidins and derivatives can stimulate proliferation of tammar wallaby mammary epithelial cells *in vitro*, and their gene expression level is elevated in mammary glands in late involution. These results suggested that the roles of cathelicidins in marsupial reproduction may not be limited to antimicrobial protection of pouch young, but also include regulation of mammary cell proliferation during lactation and mammary gland re-modeling during involution (Wanyonyi et al., 2011).

## Marsupials As A Source for Novel Antimicrobials

As discussed above, marsupials have developed multiple strategies to protect immunologically naive young. It has been hypothesized that the pathogenic pressure during early development has also led to strong selective pressures on marsupial immune genes, resulting in high degrees of duplication and diversification in certain gene families, such as antimicrobial peptides cathelicidins (Wang et al., 2011) and defensins (Jones et al., 2016).

Cathelicidins have undergone lineage-specific expansion within marsupials, giving rise to multiple genes with high sequence variability (Belov et al., 2007; Daly et al., 2008). While many eutherian mammals (e.g., primates, rodents, rabbits, and carnivores) have only one cathelicidin, the gray short-tailed opossum (*Monodelphis domestica*), tammar wallaby, and Tasmanian devil have 12, 14, and six cathelicidin genes in their genomes, respectively (Belov et al., 2007; Daly et al., 2008; Peel et al., 2016). These marsupial cathelicidins are highly variable in the mature peptide domain, showing only 3–47% pair-wise sequence similarities. Phylogenetic analysis showed that the genes form lineage or species-specific clades, suggesting that the cathelicidin gene family has been subject to multiple duplication events followed by rapid gene diversification throughout marsupial evolutionary history (Peel et al., 2016).

Similarly, marsupial defensin families have also undergone species-specific expansions, resulting in 48, 34, and 39 putative defensin genes in the Tasmanian devil, koala, and tammar wallaby, respectively (Jones et al., 2016). Among these peptides, 112 were found to exhibit characteristics required for the classical antimicrobial function, such as a cationic net charge and a high proportion of hydrophobic residues. Several functionally important codon sites within the mature peptide domain were detected to be subject to positive selection, which may have been caused by host-pathogen co-evolution (Jones et al., 2016).

A range of peptides derived from human cathelicidin or defensins are currently being investigated as novel antibiotics, though there are some common issues associated with these peptides, such as instability, hemolytic activity, and salt sensitivity (reviewed in Aoki and Ueda, 2013). The large copy number and high sequence diversity of marsupial antimicrobial peptides make them a good source for new antimicrobial discovery and peptide design. So far, 15 marsupial cathelicidin derived peptides have been tested *in vitro* for antimicrobial potential, including six Tasmanian devil peptides, eight tammar wallaby peptides, and one predicted ancestral peptide reconstructed from tammar wallaby cathelicidin sequences (Wang et al., 2011; Wanyonyi et al., 2011; Peel et al., 2016). Five of these peptides showed broad-spectrum bactericidal and fungicidal activity, while one (Saha-CATH3) was specifically potent against *Cryptococcus neoformans* fungal strains (**Table 1**). Two peptides, WAM1 and Saha-CATH5, also effectively killed antibiotic-resistant strains, such as *Pseudomonas aeruginosa*, *Klebsiella pneumoniae*, *Acinetobacter baumannii*, methicillin-resistant *Staphylococcus aureus* (MRSA), and vancomycin-resistance *Enterococcus faecalis* (VREF). Hemolytic assays demonstrated that all examined marsupial peptides are not toxic to human red blood cells except at extremely high peptide concentration (e.g., >250 μg/ml) (Wang et al., 2011; Peel et al., 2016). Salt sensitivity test of WAM1 showed that, unlike most other cathelicidins which lose activity under high salt conditions, WAM1 is resistant to inhibition by high salt concentrations (150–200 mM NaCl) (Wang et al., 2011).

**Table 1:** Antimicrobial activity of six marsupial cathelicidin peptides.

	Strains	Minimum inhibitory concentration^a^ (μM)					
		
		WAM1^b^	WAM2^b^	Ancestral WAM^b^	Saha- CATH3^c^	Saha- CATH5^c^	Saha- CATH6^c^
Gram-negative	*Escherichia coli*	0.47	1.46	0.41		13.9	
	*Pseudomonas aeruginosa*	0.77	1.29	2.06			
	*Salmonella enterica*	1.14	1.58	0.96			

Gram-positive	*Bacillus subtilis*	1.5	2.14	1.56			
	*Staphylococcus aureus*	1.01	1.39	1.42			
	*Streptococcus agalactiae*						16.9
	*Streptococcus anginosus*					13.9	16.9
	*Streptococcus oralis*/mitis group						16.9
	*Streptococcus pneumoniae*						16.9
	*Streptococcus pyogenes*	0.66	0.39	0.68		13.9	16.9
	*Streptococcus uberis*	1.22	0.63	0.07			

Fungi	*Candida krusei*						16.9
	*Candida albicans*	1.3	1.47	6.45			
	*Cryptococcus neoformans*				4.16	13.9	16.9
	*Cryptococcus gattii*						16.9

Drug-resistant isolates	Methicillin-resistant *Staphylococcus aureus*					13.9	
	Vancomycin-resistant *Enterococcus faecalis*					13.9	16.9
	*Pseudomonas aeruginosa* isolates	0.47-30.4					
	*Acinetobacter baumannii* isolates	0.95-15.2					
	*Klebsiella pneumoniae* isolates	0.95-7.59					


*^a^Only MIC values lower than 20 μM are shown. ^b^Data from Wang et al. (2011). ^c^Data from Peel et al. (2016).*

These studies are the first steps to fully revealing the potential of marsupial cathelicidins as candidates for novel antibiotic development. Further work is required to evaluate the pharmacokinetics of the peptides and to understand the mechanisms of their functions. Moreover, the issue of high cost of peptide production needs to be addressed. Past and current studies of marsupial cathelicidins largely rely on chemical synthesis of peptides, which is more expensive compared to recombinant expression approaches (van Dijk et al., 2011). Further research on peptide cytotoxicity and stability will facilitate the design and optimization of a viable expression system to enable peptide production on a larger scale. Studying of core elements that are responsible for activities will also help reduce the size of peptides to produce and thereby improve the cost-effectiveness.

## Conclusion

**FIGURE 3:**
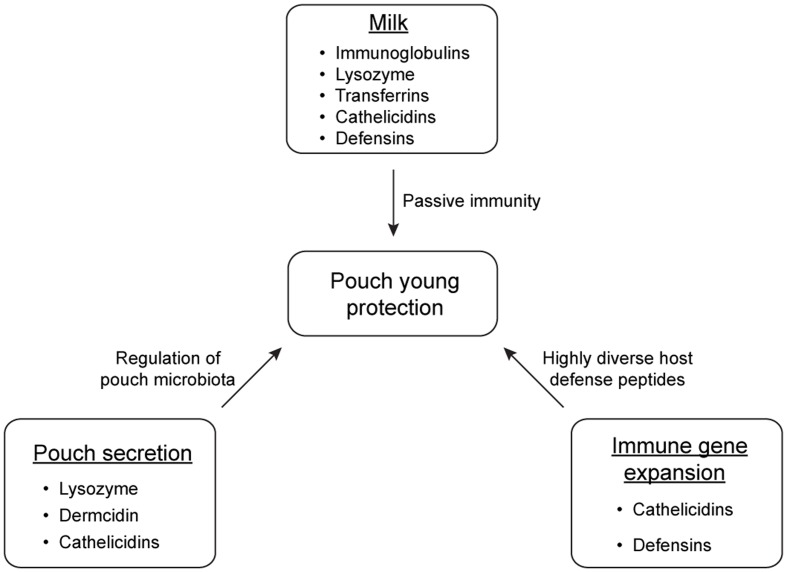
**Main strategies of antimicrobial protection of marsupial pouch young**.

Marsupials have developed multiple strategies to protect immunologically naive young in the non-sterile environment of the pouch (summarized in **Figure 3**). Pouch secretions reduce the prevalence of certain harmful microbes in the pouch during lactation, and the milk provides passive immunity for the young at key developmental stages. Immune compounds such as lysozyme, dermcidin, immunoglobulins, transferrin, and cathelicidins play crucial roles in the antimicrobial protection of marsupial pouch young. Studying protective mechanisms in the marsupial pouch will not only improve our understanding on the importance of these components in the mammalian immune system, but will also provide a unique opportunity to discover novel antimicrobials to combat fast-evolving pathogens.

## Author Contributions

YC wrote the manuscript. KB provided feedback and helped revise the manuscript.

## Conflict of Interest Statement

The authors declare that the research was conducted in the absence of any commercial or financial relationships that could be construed as a potential conflict of interest.
